# Laparoscopic ureteroplasty with buccal mucosa graft for long proximal ureteral stenosis: A step by step video

**DOI:** 10.1590/S1677-5538.IBJU.2018.0830

**Published:** 2020-01-13

**Authors:** Conrado Menegola, Patric Machado Tavares, Nelson Sivonei Batezini, Antonio Rebello Horta Gorgen, Tiago Elias Rosito

**Affiliations:** 1 Departamento de Urologia, Hospital de Clínicas de Porto Alegre, Santa Cecília, Porto Alegre, RS, Brasil

## Abstract

**Introduction:**

Strictures of the proximal ureter may occur from iatrogenic injury or impacted kidney stones. Many methods of surgical correction have been described, but the current treatment options can have significant morbidity associated with bowel substitution and vascular complications. Buccal mucosa grafting (BMG) has revolutionized the management of urethral strictures, but has not gained widespread use for ureteroplasties. Naude first described the use of BMG in the open repair of ureteral strictures, but only recently new techniques with minimally invasive approaches are being described.

**Objective:**

To show in this video the step-by-step of a laparoscopic inlay ureteroplasty with buccal mucosa grafting of a 75-year-old male with a long left proximal ureteral stricture secondary to previous ureteroscopies.

**Patient and Methods:**

A healthy 75-year-old male with history of two previous ureteroscopies for impacted left proximal ureteral stones, presented with left lumbar pain. Urinary ultrasound and computed tomography showed left hydronephrosis. Pyelography revealed a long left proximal ureteral stenosis. The patient was submitted to a laparoscopic ureteroplasty with BMG after incision of the anterior aspect of the stenotic ureteral segment. The surgery was 120 minutes long, with no complications. The patient was discharged on second post-operative day and the ureteral stent was removed after 4 weeks, with a clinical and radiological success.

**Conclusion:**

This technique is an excellent option that has been described recently, with low morbidity and good results. It offers the possibility of resolving long proximal ureteral strictures precluding the need for more extensive and morbid surgeries.

## ARTICLE INFO

**Available at: http://www.intbrazjurol.com.br/video-section/20180830_Menegola_et_al**

**Int Braz J Urol. 2020; 46 (Video #4): 141-142**

